# Shared Dynamics of Food Decision-Making in Mother-Child Dyads

**DOI:** 10.3389/fpsyg.2021.695388

**Published:** 2021-08-12

**Authors:** Oh-Ryeong Ha, Amanda S. Bruce, Haley J. Killian, Ann M. Davis, Seung-Lark Lim

**Affiliations:** ^1^Department of Psychology, University of Missouri–Kansas City, Kansas City, MO, United States; ^2^Department of Pediatrics, University of Kansas Medical Center, Kansas City, KS, United States; ^3^Center for Children's Healthy Lifestyles & Nutrition, Kansas City, MO, United States

**Keywords:** eating decisions, weight status, eating behavior, children, mother-child dyad, obesity

## Abstract

This study explored risk parameters of obesity in food decision-making in mother-child dyads. We tested 45 children between 8–12 years and their biological mothers to measure the decision weights of food health attributes, the decision weights of food taste attributes, self-regulated food decisions, and self-reported self-control scores. Maternal body mass index (BMI), and children's BMI-percentiles-for-age were also measured. We found a positive correlation between children's and their mothers' decision weights of taste attributes in food decision-making. We also found a positive correlation between children's BMI %iles and their mothers' BMIs. Children with overweight/obesity demonstrated lower correlations between health and taste ratings and a lower percentage of self-regulated food decisions (i.e., resisting to eat tasty but unhealthy foods or choosing to eat not-tasty but healthy foods) than children with healthy weight. Our findings suggested that the decision weights of taste attributes and weight status shared similar patterns in mother-child dyads. Also, the findings suggested that establishing dynamics of unhealthy food-decision making may increase the risk of childhood obesity. Helping children to develop the dynamics of healthy food-decision making by increasing the importance of health while decreasing the importance of taste may promote resilience to susceptibility to unhealthy eating and weight gain.

## Introduction

Obesity is a significant health condition that costs over $190 billion of healthcare spending in the U.S. (Cawley and Meyerhoefer, 2012). The prevalence of obesity has risen for the past several decades (Ogden et al., 2016). Recent data shows that 39.8% of adults and 18.5% of youth between ages 2–19 are obese (Hales et al., 2017). Trends predict that about half of adults, one in three adolescents between ages 12–19, and one in four children between ages 6–11 will be obese by 2030 (Wang et al., 2020). The increasing epidemic is linked to the modern obesogenic environment, including the lower cost for energy-dense fast foods, unhealthy food marketing, and increased portion sizes, which perpetuates individuals' unhealthy eating behaviors (Rodgers et al., 2018). Considering that we make numerous eating decisions daily, in various contexts and conditions (Sobal and Bisogni, 2009), how well an individual establishes healthy food decision-making would significantly contribute to healthy eating and weight management.

For children, the relationships between unhealthy food decision-making and the risk of weight gain should be understood within parent-child relationships. Food intake is highly parent-dependent until adolescence because parents typically select and provide foods for children, and children observe and model parents' eating behaviors. Parental obesity and unhealthy food decision-making reflect the genetic and environmental risks of developing obesity (Savage et al., 2007). Parental obesity is related to the high likelihood of obesity in their offspring (Grilo and Pogue-Geile, 1991; Whitaker et al., 1997; Pachucki et al., 2014; Shearrer et al., 2018). Children's weight gain and parental BMIs show positive correlations (Grilo and Pogue-Geile, 1991; Whitaker et al., 1997). Parental preferences for palatable unhealthy foods influence children's preferences for high-fat, high-sugar, and low-nutrient unhealthy foods and eating behaviors (Fisher and Birch, 1995; Wardle et al., 2001; Benton, 2004; Ventura and Birch, 2008). Heightened susceptibility to tasty, unhealthy foods increases reward-motivated hedonic eating. In both adults and children with overweight, obesity or high body fat%, food cues with high sugar and fat strongly activate the reward and taste processing signals in the brain that lead to overeating and weight gain (Davis et al., 2007; Stice and Yokum, 2016; Bohon, 2017). A neuroimaging study revealed that food cues with high sugar and fat activate reward systems in the brain greater in children of obese parents than those of healthy weight parents (Shearrer et al., 2018). Overall, these findings suggest that weight gain and susceptibility to tasty unhealthy foods of children and their parents could increase the risks of obesity in children. Still, further investigation is needed to determine how susceptibility to tasty unhealthy foods would be linked to weight gain in children. For this line of investigation, the present study aimed to investigate the development of children's food decision-making and self-control in the context of parent-child dyads.

One key element for establishing the dynamics of healthy food decision-making and weight management is self-control. Unlike high self-controllers who incorporate both health and taste attributes, low self-controllers mainly incorporate taste attributes in food choices (Hare et al., 2009). Low self-controllers demonstrate a delay in incorporating health attributes in food choices compared to high self-controllers (Sullivan et al., 2015). The dynamics of food decision-making are similar across low self-controllers and adults with overweight/obesity. Adults with overweight/obesity often fail to make self-regulated food decisions (Fan and Jin, 2014; Lim et al., 2018). Adults with overweight/obesity exercise lower levels of self-control than adults with healthy weight (Fan and Jin, 2014). Even if adults with any weight status incorporate taste attributes more than health attributes for food choices, adults with overweight incorporate health attributes less into their food choices, similar to low self-controllers (Lim et al., 2018).

Similar to adults, children's self-control capacity is related to dynamics of healthy food decision-making (van Meer et al., 2017; Ha et al., 2019). Although the utility of self-control is limited in children given prolonged development until early adulthood (Diamond, 2013), emerging self-control capacity is a good predictor of later successful self-control (Moffitt et al., 2011). It was found that emerging dietary self-control is involved in the healthy eating decision-making process when children resist the predominant response of eating tasty but unhealthy foods (Ha et al., 2016). However, children's developing control systems are less effective in incorporating health attributes in food choices compared to adults (van Meer et al., 2017). Moreover, we previously found that children's lower self-control is linked to a decreased association between food healthiness and tastiness, which reflects a stronger tendency of processing unhealthy food tasty (Ha et al., 2019). The weaker association between healthiness and tastiness may prompt unhealthy eating decisions that lead to overeating and weight gain considering that most children primarily incorporate taste attributes while ignoring health attributes in food choices (Bruce et al., 2016; Lim et al., 2016; Ha et al., 2018). Yet, it needs to be confirmed how susceptibility to tasty unhealthy food represented by the association between healthiness and tastiness is related to self-regulated food decisions in children.

Children's self-control is also related to weight status (Nederkoorn et al., 2006; Batterink et al., 2010). Children with obesity are more likely to utilize lower levels of self-control, which often links to overeating and low responsiveness to obesity intervention (Nederkoorn et al., 2006). Low self-controllers during early childhood are likely to gain more weight, more quickly in later childhood and adolescence (Anzman and Birch, 2009; Francis and Susman, 2009). Neuroimaging studies found supporting evidence to behavioral findings. Whereas unhealthy food cues increase activation in the control systems in children with healthier weight (van Meer et al., 2016), those cues increase activation in the reward systems and decrease activation in the control systems in children with obesity (Batterink et al., 2010).

For establishing the dynamics of healthy food decision-making in children, parental guidance is crucial. In children, whereas food choices based on children's own food preferences activate the reward systems, food choices based on projected maternal food selections for them activate the control systems, which suggests that children make more self-regulatory eating decisions when they are externally cued by considering maternal choices for them (Lim et al., 2016). However, if parents and children fail to successfully utilize self-control in food decision-making, it may result in unhealthy eating habits throughout the family. Children's obesity is associated with parental lower self-control, and both parents and children who exercise lower self-control are more likely to develop obesity (Stoklosa et al., 2018). This suggests the strong association between poorly self-regulated unhealthy eating and the risk of obesity in parent-child dyads (Stoklosa et al., 2018). Yet, how the dynamics of unhealthy food decision-making are *shared* between parents and children is still unanswered.

The present study aimed to examine risk parameters of obesity in children by exploring the dynamics of unhealthy food decision-making and weight status in biological mother-child dyads. We expected that dynamics of unhealthy food decision-making would be indicated by significant relations among (1) lower decision weights of healthiness attributes, (2) higher decision weights of taste attributes, and (3) weaker associations between food healthiness and tastiness (i.e., negative correlations between health and taste ratings), and/or (4) lower percentages of self-regulated food decisions along with lower self-control capacity within children and mothers. Importantly, we hypothesized that mother-child dyads would demonstrate similar dynamics of food decision-making. Lastly, we hypothesized that children with overweight/obesity would show dynamics of unhealthier food decision-making than children with healthy weight.

## Materials and Methods

### Participants

The sample of this study consisted of 45 children (27 girls, 18 boys) aged 8 to 12 years (*M* = 10.4 years, *SD* = 1.5) who spoke English as their first language, and their biological mothers (*M* = 38.9 years, *SD* = 5.9), who communicated in English fluently for understanding experiment directions and survey questions. Families were recruited using flyers distributed to schools, hospitals, and community centers in Kansas City metropolitan and nearby rural areas. In addition, flyers were posted on social media, or sent with a letter to parents of prospective child participants who agreed to be contacted for future research studies at the University of Kansas Medical Center identified using HERON database (Healthcare Enterprise Repository for Ontological Narration) (Waitman et al., 2011). The sample included healthy participants with normal or corrected-to-normal vision and hearing without a history of neurological disorders, clinically significant psychopathology, or learning disabilities. Children consisted of 25 White (55.6%), 11 Multiracial (24.4%), five Hispanic or Latina/o (11.1%), and four Black or African American (8.9%). Mothers consisted of 34 White (75.6%), six Hispanic or Latina (13.3%), and five Black or African American (11.1%).

To assess the body mass index (BMI; kg/m^2^), we measured participants' heights and weights using a Perspective Enterprises standard stadiometer (PE-WM-60-84; Portage, Michigan) and a Befour scale (PS6600 ST; Saukville, Wisconsin) in light clothing without shoes. We computed each child's BMI percentile-for-age that considers a weight for height, age, and gender using the Baylor College of Medicine calculator (https://www.bcm.edu/bodycomplab/BMIapp/BMI-calculator-kids.html). The mean BMI percentile-for-age was 63.4 (*SD* = 31.8; range 5.8–99.3). When considering the weight status category, there were 29 children with healthy weight (20 girls, nine boys; *M*_*age*_= 10.3, *SD* = 1.5; *M*_*BMI*−*Percentile*_= 45.4, *SD* = 25.4), and 16 children with overweight/obesity (four overweight, 12 obesity; seven girls, nine boys; *M*_*age*_= 10.6, *SD* = 1.5; *M*_*BMI*−*Percentile*_ = 95.9, *SD* = 2.6). The mean BMI for mothers was 30.0 (*SD* = 7.0; range 17–46). There were 12 mothers with an underweight or healthy weight (two underweight; *M*_*BMI*_ = 21.5, SD = 2.5) and 33 mothers with overweight or obesity (11 overweight; *M*_*BMI*_ = 33.1, *SD* = 5.4). We excluded data from one additional mother-child dyad before data analysis for the computation of decision weights was impossible because the child responded to food rating trials with the same response throughout all trials.

Mothers gave written informed consent and children gave written assent upon arrival before participation. All the procedures of this study were approved by the University of Kansas Medical Center's Huan Subjects Committee, and a request to rely was approved by the University of Missouri—Kansas City's Institutional Review Board.

### Procedure

#### Food Rating Tasks

**Figure 1:**
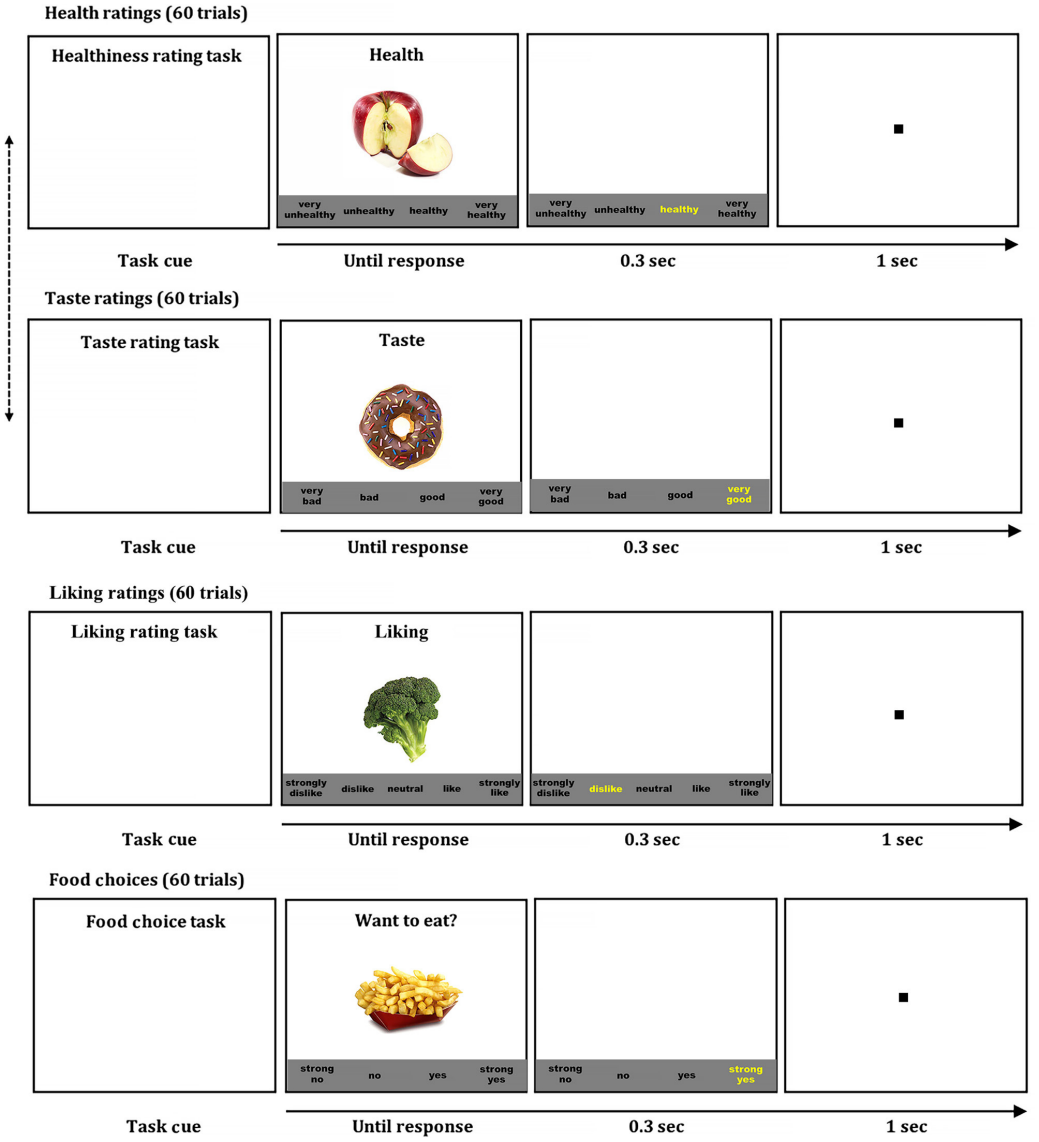
Food ratings and choice tasks. Examples of food healthiness, taste, and liking ratings, and choice tasks. Pressing a space bar presented a colored food image on the screen, which was remained until a response. The order of food items was randomized in each tasks, and the order of health and taste ratings were counterbalanced across participants.

A mother and a child were tested using the same tasks in separate rooms. We used computerized food rating and choice tasks to measure an individual's perceived food healthiness, taste, and liking, and food choices (see Figure 1) (Bruce et al., 2016; Lim et al., 2016; Ha et al., 2018, 2019, 2020). For maintaining adequate hunger levels for realistic food choices, participants were instructed to fast for 2 h before coming to the laboratory. First, participants rated food healthiness and taste separately on 60 food items that were consisted of 30 healthy foods (e.g., vegetables, fruits, and legumes) and 30 unhealthy foods (e.g., processed meats, fried foods, and sweet foods). Participants rated each food-related attribute using multiple-point scales in ascending or descending order, which was maintained consistently across rating tasks within a participant. Participants were instructed to rate food items on the healthiness regardless of the taste and the food taste regardless of the healthiness in a separate block. The order of health and taste ratings was counterbalanced across participants. The presentation order of food items was randomized within each rating task. Each attribute was rated using a 4-point scale from “very unhealthy” to “very healthy” (or from “very healthy” to “very unhealthy”) for the healthiness, and from “very bad” to “very good” for the taste. Then participants rated food liking using a 5-point scale from “strongly dislike” to “strongly like.” Lastly, participants made food choices (“Do you want to eat?”) for each food item using a 4-point scale from “strong no” to “strong yes.” At the beginning of each task, the instruction was displayed on the computer screen to specify which attribute participants would rate. After a fixation point (1s), one of 60 colored food images (72 dpi, 300 x 300 pixels) in a white background was presented in the center along with the response options in the black text below the food image. The food image and response options remained on the screen until a participant pressed a key on a keyboard to select a response. The selected response was highlighted in yellow briefly for visual feedback. We used Presentation® (version 20; Neurobehavioral Systems, Berkley, California; RRID: SCR_002521) for the stimulus presentation and response collection. To warrant motivation for realistic food choices, participants were instructed that they would randomly receive one of the food items they chose to eat (yes or strong yes) in the choice task, and they received one food item after completing the task.

#### Questionnaire

Participants completed the Self-Control Scale (Tangney et al., 2004) to report their perceived levels of self-control. This scale has been used for adults as well as children and adolescents (Duckworth et al., 2010; Ha et al., 2019). This scale consists of 36 statements regarding self-control-related characteristics (only one food-related self-control item). Items include “I change my mind fairly often,” “I keep everything neat,” and “I'd be better off if I stopped to think before acting.” For answering any questions, our research staff stayed with child participants while they were completing questionnaires. Participants were asked to indicate how much each statement reflects their characteristics using a 5-point scale from “not at all” to “very much.” The self-control scores range between 36 and 180, and higher scores indicate higher amounts of self-control. The mean score was 126.2 (range = 91–151) for children, and 134.5 (range = 112–154) for mothers (see Table 1). Cronbach's alphas for children and mothers were 0.773 and 0.765 in this study, respectively.

**Table 1:** Descriptive statistics.

		**Children**		**Mothers**	
		**M**	**SD**	**M**	**SD**
BMIs (Percentiles for children)		63.4	31.8	30.0	7.0
Estimated health β Coefficients		−0.003	0.21	0.15	0.16
Estimated taste β Coefficients		0.93	0.30	1.07	0.28
Correlations between health and taste		−0.13	0.25	0.16	0.35
Self-control scores		126.2	14.2	134.5	11.4
Self-regulated decisions (%)		15.1	14.5	29.0	18.0
	*unhealthy/tasty/no* (freq)	3.87	3.88	7.04	4.59
	*healthy/not-tasty/yes* (freq)	0.95	1.19	0.69	1.08
Taste ratings	Unhealthy foods	3.30	0.40	2.94	0.54
	Healthy foods	2.98	0.37	3.14	0.31
Health ratings	Unhealthy foods	1.94	0.40	1.73	0.27
	Healthy foods	3.30	0.36	3.62	0.13
Liking ratings	Unhealthy foods	3.90	0.54	3.54	0.60
	Healthy foods	3.53	0.48	3.91	0.40
Choices	Unhealthy foods	3.01	0.45	2.58	0.52
	Healthy foods	2.73	0.37	2.98	0.35

### Statistical Analyses

We adopted our previous statistical analysis model (Bruce et al., 2016; Lim et al., 2016; Ha et al., 2019, 2020) that estimated the decision weights of food health and taste attributes in food decision-making. We fitted a linear regression model that taste and health ratings simultaneously predicted food liking ratings at the individual level. The regression beta coefficients of health and taste attributes indicated the relative decision weights of food healthiness and taste in food preferences, respectively.

To estimate the association between food healthiness and tastiness, we computed a Pearson's correlation coefficient between healthiness and taste ratings of 60 food items in each individual (Ha et al., 2019). The larger the positive correlation coefficient of health and taste ratings, the stronger the association between food healthiness and tastiness (i.e., “*healthy* = *tasty*” association). The larger the negative correlation coefficient of health and taste ratings, the stronger the association between food unhealthiness and tastiness (i.e., “*unhealthy* = *tasty*” association). To note, the health or taste beta coefficient and the correlation between health and taste ratings are independent constructs, while the formers estimate the decision weight of health or taste ratings in food decisions and the latter indicates individuals' perceived association between food healthiness and tastiness.

Further, we computed the percentages of self-regulated food decisions (Ha et al., 2016, 2020; Lim et al., 2016). First, we identified food items that each participant assessed as *tasty* (i.e., “good” or “very good” ratings) or *not-tasty* (“bad” or “very bad” ratings) for 30 healthy and 30 unhealthy food items separately. Next, we categorized food items as *unhealthy/tasty, unhealthy/non-tasty, healthy/tasty*, and *healthy/non-tasty*. Then, we classified the self-regulated food decisions that needed self-control to make healthier choices when individuals resisted the temptation of eating tasty but unhealthy food items successfully (i.e., *unhealthy/tasty/no*: “no” or “strong no” decisions on *unhealthy/tasty* food items) or when individuals chose to eat not-tasty but healthy food items successfully (i.e., *healthy/non-tasty/yes*: “yes” or “strong yes” decisions on *healthy/non-tasty* food items). Lastly, we computed the percentages of self-regulated decisions out of *unhealthy/tasty* and *healthy/not-tasty* food items.

Spearman's rho correlations were used to explore relationships across variables in children, mothers, and mother-child dyads. Mann-Whitney *U* tests were used to compare the dynamics of food decision-making across children of different weight statuses.

## Results

### Dynamics of Food Decision-Making

#### Children

Descriptive statistics are listed in Table 1. To examine the dynamics of unhealthy food decision-making in children, we first conducted Spearman's rho correlational analyses across the BMI-percentile-for-age, the health beta coefficient, the taste beta coefficient, the correlation between health and taste ratings, the self-control score, and the percentage of self-regulated decision (see Table 2). As the correlation between health and taste ratings decreased, the health beta coefficient decreased in food preferences, *r*(43) = 0.50, *p* < 0.001. As the BMI-percentile-for-age increased, the percentage of self-regulated decisions decreased, *r*(43) = −0.31, *p* = 0.042. As the correlation between health and taste ratings decreased, the percentage of self-regulated decisions decreased, *r*(43) = 0.63, *p* < 0.001. These results suggest that children are less likely to incorporate health attributes in food decision-making as the “*unhealthy* = *tasty*” association is stronger. Furthermore, children are more likely to make poorly self-regulated unhealthy decisions as the “*unhealthy* = *tasty*” association is stronger and the BMI is higher.

**Table 2:** Correlations for dynamics of unhealthy food decision-making in children.

	**1**	**2**	**3**	**4**	**5**	**6**
1. Children's BMI-percentiles-for-age	–					
2. Children's estimated *health β* coefficients	−0.24	–				
3. Children's estimated *taste β* coefficients	0.05	−0.25	–			
4. Children's correlations between health and taste ratings	−0.25	0.50^***^	−0.11	-		
5. Children's self-control scores	−0.18	0.06	0.09	0.17	–	
6. Children's percentages of self-regulated decisions	−0.31^*^	0.28	−0.17	0.63^***^	0.08	–

*Spearman's rhos are reported on diagonals for scale measures.*

* *p < 0.05,*

*** *p < 0.001*.

#### Mothers

Similar to children's data, we conducted Spearman's rho correlational analyses across the BMI, the health beta coefficient, the taste beta coefficient, the correlation between health and taste ratings, the self-control score, and the percentage of self-regulated decision (see Table 3). As the correlation between health and taste ratings decreased, the beta coefficient of the taste increased in food preferences, *r*(43) = −0.41, *p* = 0.006. As the self-reported self-control score decreased, the percentage of self-regulated decisions decreased, *r*(43) = 0.37, *p* = 0.013. These results suggest that mothers incorporate taste attributes more in food decision-making as the “un*healthy* = *tasty*” association is stronger. Additionally, lower self-controllers are more likely to make poorly self-regulated unhealthy decisions.

**Table 3:** Correlations for dynamics of unhealthy food decision-making in mothers.

	**1**	**2**	**3**	**4**	**5**	**6**
1. Mothers' BMIs	–					
2. Mothers' estimated *health β* coefficients	−0.09	–				
3. Mothers' estimated *taste β* coefficients	0.08	−0.39^**^	–			
4. Mothers' correlations between health and taste ratings	−0.09	0.07	−0.41^**^	–		
5. Mothers' self-control scores	0.10	0.27	0.01	0.26	–	
6. Mothers' percentages of self-regulated decisions	0.03	0.22	−0.18	0.25	0.37^**^	–

*Spearman's rhos are reported on diagonals for scale measures.*

** *p < 0.01*.

#### Mother-Child Dyads

To explore the similarity in the dynamics of food decision-making in mother-child dyads, we conducted Spearman's rho correlational analyses across the aforementioned variables of mothers and those of children (see Table 4). It was revealed that as the BMIs of mothers increased, the BMI-percentiles of children increased as well, *r*(43) = 0.30, *p* = 0.043. Also, as mothers' taste beta coefficients increased in food preferences, children's taste beta coefficients increased in food preferences, *r*(43) = 0.33, *p* = 0.029. In addition, as mothers' self-regulated food decisions decreased, children's taste beta coefficients increased, *r*(43) = −0.30, *p* = 0.043. These results suggest that the weight status and the decision weights of food taste attributes in food decision-making show similar trends in mother-child dyads. Also, it suggests that children are more likely to have higher importance of the taste in food decision-making as mothers make poorly self-regulated food decisions.

**Table 4:** Correlations for dynamics of unhealthy food decision-making in mother-child dyads.

	**7. Mothers' BMIs**	**8. Mothers' estimated *health β* coefficients**	**9. Mothers' estimated *taste β* coefficients**	**10. Mothers' correlations between health and taste ratings**	**11. Mothers' self-control scores**	**12. Mothers' percentages of self-regulated decisions**
1. Children's BMI-percentile-for-age	0.30^*^	0.07	0.05	−0.20	−0.09	0.14
2. Children's estimated *health β* coefficients	0.11	0.12	−0.04	0.13	0.20	0.04
3. Children's estimated *taste β* coefficients	−0.05	−0.25	0.33^*^	0.13	−0.11	−0.30^*^
4. Children's correlations between health and taste ratings	0.06	0.03	−0.18	0.14	−0.16	−0.04
5. Children's self-control scores	−0.24	−0.21	0.06	−0.06	−0.06	−0.17
6. Children's percentages of self-regulated decisions	−0.06	0.01	−0.11	0.17	−0.21	−0.02

*Spearman's rhos are reported.*

* *p < 0.05*.

### Weight Status and Dynamics of Food Decision-Making

**Figure 2:**
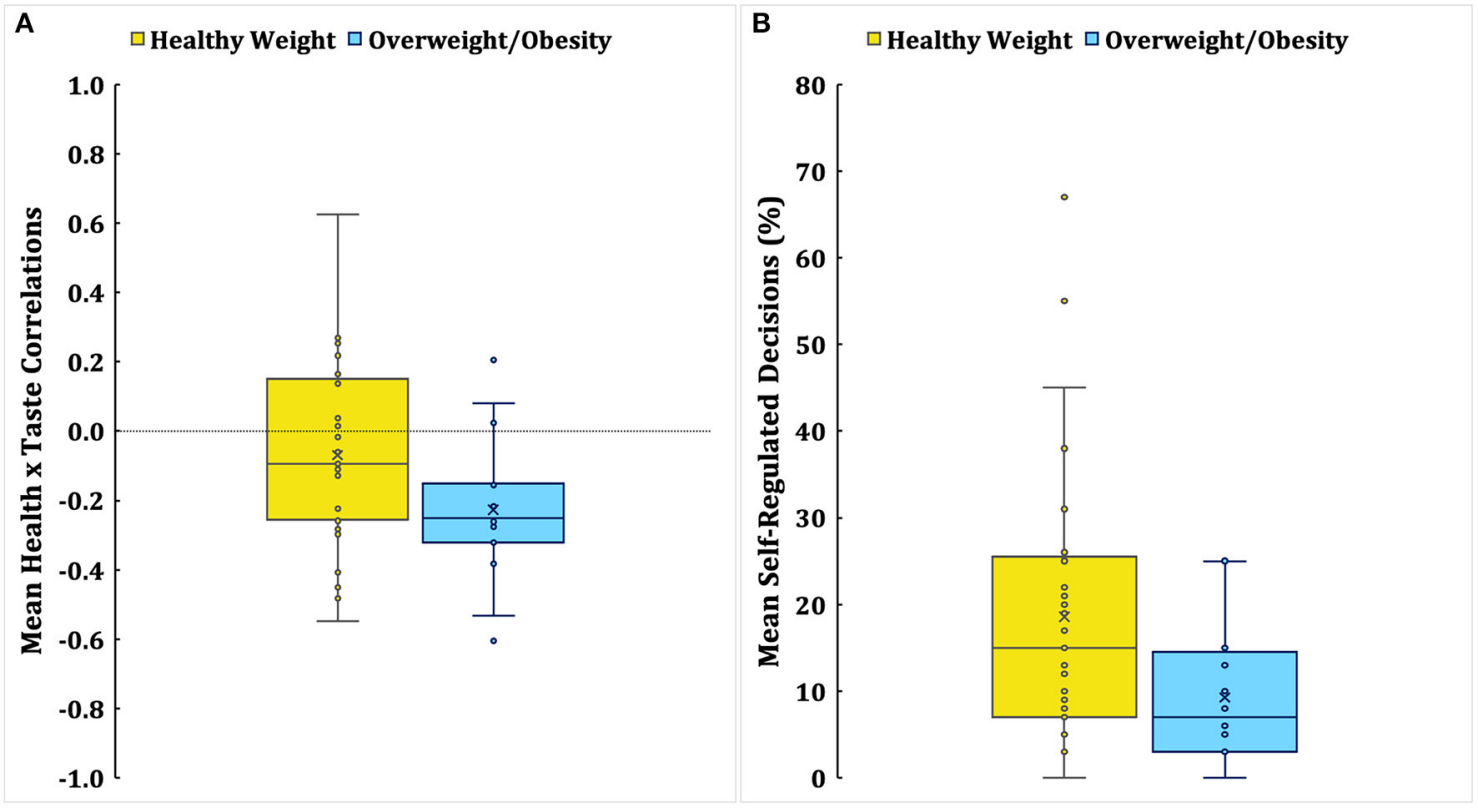
Mean correlations between health and taste ratings (**A**) and percentages of self-regulated decisions (**B**) in children by weight status. An X mark indicates the mean, and a horizontal line within a box indicates the median. The dotted line in **(A)** illustrates the zero correlation point.

We conducted Mann-Whitney *U* tests to explore whether the dynamics of food decision-making would be different between children with healthy weight and children with overweight/obesity (see Table 5). Compared to children with healthy weight, children with overweight/obesity had significantly lower correlations between health and taste ratings, *Z* = −2.02, *p* = 0.044, and lower percentages of self-regulated decisions, *Z* = −2.14, *p* = 0.033 (see Figure 2). Children's health and taste beta coefficients, self-control scores, or other mother-related variables were not significantly different between groups. These results suggest that children with overweight/obesity demonstrated the dynamics of unhealthier food decision-making given the stronger “*unhealthy* = tasty” association, and poorly self-regulated food decisions compare to children with healthy weight.

**Table 5:** Dynamics of food decision-making by children's weight statuses.

		**Children healthy weight**		**Children overweight/obesity**		**Mann-Whitney** ***U***	
		**M**	**SD**	**M**	**SD**	***Z***	***p***
Children	BMI-Percentile-for-age	45.4	15.4	95.9	2.6	–**5.50**	** < 0.001**
	Estimated Health β Coefficients	0.03	0.24	−0.06	0.13	−1.76	0.079
	Estimated Taste β Coefficients	0.93	0.33	0.92	0.25	−0.12	0.906
	Correlations between Health and Taste Ratings	−0.07	0.27	−0.23	0.21	**-2.02**	**0.044**
	Self-Control Scores	127.2	13.1	124.2	16.2	−0.18	0.859
	Self-Regulated Decisions %	18.5	16.3	9.0	7.7	–**2.14**	**0.033**
Mothers	BMIs	28.4	4.8	32.8	9.4	−1.83	0.068
	Estimated Health β Coefficients	0.16	0.16	0.13	0.17	−0.57	0.569
	Estimated Taste β Coefficients	1.03	0.32	1.14	0.18	−0.85	0.393
	Correlations between Health and Taste Ratings	0.23	0.38	0.02	0.24	−1.87	0.061
	Self-Control Scores	134.9	10.4	133.8	13.5	−0.21	0.831
	Self-Regulated Decisions %	28.8	19.0	29.6	16.6	−0.18	0.859

*Bold values represent statistically significant findings*.

## Discussion

The current study investigated similarities of dynamics of food decision-making in mother-child dyads to explore the risk parameters of obesity in children. Particularly, we explored the associations across the decision weights of healthiness attributes, the decision weights of taste attributes, the associations between food healthiness and tastiness, self-regulated food decisions, self-control capacity, and weight status within and across children and mothers. For children, we found that children who had the stronger “*unhealthy* = tasty” association were less likely to incorporate health attributes in food preferences. This suggests that forming the stronger “*unhealthy* = tasty” association, in other words, processing unhealthy foods taste better, would be related to the decreased importance of food healthiness in children's eating decisions. For children, taste information plays a pervasive role in children's food liking and eating decisions, while health information is considered to a lesser degree (Cornwell and McAlister, 2011; Ha et al., 2019). Thus, a strong tendency of perceiving unhealthy foods as tasty would coincide with the further reduced importance of food healthiness. Also, children who had the stronger “*unhealthy* = tasty” association were less likely to make self-regulated food decisions. It suggests that a strong tendency of perceiving unhealthy foods as tasty would be linked to poorly self-regulated unhealthy food decisions. Moreover, children who had higher BMI-percentiles-for-age were less likely to make self-regulated decisions. It supports our hypothesis that predicted the relationship between higher BMIs and poorly self-regulated food decisions.

For mothers, we found that mothers who had the stronger “*unhealthy* = tasty” association were more likely to have the higher decision weight of taste attributes in food decision-making as hypothesized. It suggests that forming the stronger “*unhealthy* = tasty” association would increase the importance of the taste in eating decisions. Adults tend to relatively rely less on the sole taste information in food decision-making than children. However, a strong tendency of perceiving unhealthy foods as tasty would be linked to the heightened importance of taste in eating decisions. Additionally, mothers with lower self-reported self-control scores were more likely to make poorly self-regulated food decisions, which supports our hypothesis that lower self-control capacity is linked to poorly self-regulated unhealthy food decisions.

In mother-child dyads, we found similarities in the dynamics of unhealthy food decision-making. Maternal and children's BMIs were significantly positively correlated. This result supports that children are more likely to have a higher risk of weight gain as parental BMIs increase, as other studies have demonstrated (Grilo and Pogue-Geile, 1991; Whitaker et al., 1997). Regarding dynamics of food decision-making, the decision weights of taste attributes were significantly positively correlated between mothers and children. As the decision weights of taste attributes increased in children, their mothers were more likely to make poorly self-regulated food decisions. Strongly taste-oriented unhealthy eating has been identified as a contributing factor to overeating and obesity in children of parents with overweight/obesity (Fisher and Birch, 1995; Wardle et al., 2001). Further, our previous research identified that the decision weight of taste attributes mainly drives children's food decisions (Bruce et al., 2016; Ha et al., 2016; Lim et al., 2016). The intervention effect targeting the reduction of children's susceptibility to unhealthy eating was indicated by the decreased decision weight of taste attributes (Ha et al., 2018, 2020). The present finding suggests that mother-child dyads show similar trends in the degree of food taste importance in food decisions, which reflects a shared dynamic of taste-oriented food decision-making in mother-child dyads that could contribute to a higher risk of developing obesity in children.

Lastly, we compared risk parameters of obesity between children in different weight statuses. It was revealed that children with overweight/obesity showed dynamics of unhealthier food decision-making compared to children with healthy weights. As hypothesized, children with overweight/obesity were less likely to make self-regulated food decisions than children with healthy weight. Poorly self-regulated food decisions reflect that children with overweight/obesity may be more likely to have an inefficient dietary self-control mechanism that fails to resist the immediate rewards of eating tasty foods instead of considering long-term health benefits. Furthermore, the “*unhealthy* = tasty” association was stronger in children with overweight/obesity than children with healthy weight. The stronger “*unhealthy* = tasty” association increases the susceptibility to tasty unhealthy foods and reward-motivated hedonic eating (Raghunathan et al., 2006; Ha et al., 2019), which could increase the risk of obesity.

Taken together, we found profiles of food decision-making shared within and across mothers and their children. The significant correlations between mothers and children in weight status and the decision weights of taste attribute in food decision-making suggested the shared risks of excessive weight gain and taste-oriented food decisions in mother-child dyads. Previous findings of obesity treatment effects demonstrate the higher genetic risk of parental obesity, and suggest the interaction between obesity and healthy eating behaviors. The weight loss effect is less likely to be maintained in children with obese parents compared to children with nonobese parents (Epstein et al., 1987). However, the weight loss effect for both children and parents with overweight or obesity is more likely to be maintained when unhealthy food consumptions decrease in parent-child dyads (Best et al., 2016). These findings suggest that establishing dynamics of healthy food decision-making could enhance resilience to the risk of developing obesity in children.

In fact, we found that children with overweight/obesity show dynamics of unhealthier food decision-making that support the importance of establishing dynamics of healthy food decision-making. Children with overweight/obesity emphasized the taste of unhealthy foods and made poorly self-regulated unhealthy food decisions. However, we did not find differences in maternal factors in children of different weight statuses. Thus, the implication of this study should be interpreted with caution related to how maternal decision-making parameters significantly contributed to children's weight status. Nonetheless, the present study found that weight status and the decision weights of taste attributes were shared in mother-child dyads, and the weaker association between food healthiness and tastiness and poorly self-regulated food decisions were identified as important risk parameters within children and mothers. Thus, strategies targeting to reduce the importance of the taste by decreasing “*unhealthy* = *tasty*” associations or promoting more health-oriented self-regulated food decisions in the family context should be emphasized in the prevention and intervention of obesity.

This study has several limitations. The sample size was relatively modest, and the sample size was unequal across weight status given a natural sampling method. However, a weight status composition of our natural sample consisted of 35.5% of children with overweight/obesity is very similar to the prevalence of overweight or obesity in US children ages 6–11 years (34.2%) and 12–19 years (34.5%) (Ogden et al., 2014). Furthermore, our sample included 44.4% of racial and ethnic minority children that increased the generalizability of study results. To confirm how parental obesity and unhealthy eating interact to increase the risk of obesity in the offspring, future studies should explore risk parameters of obesity in a larger sample with similarly distributed sample sizes across weight statuses in mother-child dyads. The results of dynamics of food decision-making were based on correlational analyses without corrections, thus findings should be interpreted with caution, and further replications should be warranted. This study only recruited biological mothers to identify obesity risk factors in children, which excluded and limited paternal influences. Future studies should include maternal and/or paternal data for identifying food decision-making development in the family context. In addition, future studies should examine how sociodemographic backgrounds would influence the dynamics of food decision-making. The actual dietary patterns in real life were not collected in this study, and future studies should consider collecting these to improve ecological validity. Future studies should also examine how self-control would be influenced by emotional states or stress shared in mother-child dyads. Lastly, future studies could address how the intervention targeting to promote dynamics of healthy food-decision making by reducing the importance of the taste or increasing the importance of the healthiness would enhance self-regulated food decisions in children with high and low risk of developing obesity.

The findings of this study identify that mothers and children show similar taste-oriented unhealthy food decision-making dynamics that could increase the risk of obesity in children. Moreover, the findings of this study confirm that children with overweight/obesity engage in poorly self-regulated food decisions incorporating mostly taste attributes of unhealthy foods while ignoring health attributes of foods in food decision-making. The current findings imply that family-based obesity prevention and intervention approaches that focus on establishing healthier dynamics of food-decision making could be effective in reducing the prevalence of childhood obesity.

## Data Availability Statement

The raw data supporting the conclusions of this article will be made available by the authors, without undue reservation.

## Ethics Statement

This study was carried out following the recommendations of the Human Subjects Committee at the University of Kansas Medical Center and the Institutional Review Board at the University of Missouri-Kansas City. The protocol was approved by the Human Subjects Committee at the University of Kansas Medical Center. All participants gave written informed consent following the Declaration of Helsinki. All parents of participants in this study gave written informed consent, and all children gave written assent.

## Author Contributions

O-RH, AB, and S-LL contributed to the conception and design of the study. O-RH and HK organized the database. HK collected the data. O-RH and S-LL performed the statistical analysis. O-RH wrote the first draft of the manuscript. All authors contributed to manuscript revision, read, and approved the submitted version.

## Author Disclaimer

The contents are solely the responsibility of the authors and do not necessarily represent the official views of the NIH or NCATS.

## Conflict of Interest

The authors declare that the research was conducted in the absence of any commercial or financial relationships that could be construed as a potential conflict of interest.

## Publisher's Note

All claims expressed in this article are solely those of the authors and do not necessarily represent those of their affiliated organizations, or those of the publisher, the editors and the reviewers. Any product that may be evaluated in this article, or claim that may be made by its manufacturer, is not guaranteed or endorsed by the publisher.
